# Intranasal Liposomal Formulation of Spike Protein Adjuvanted with CpG Protects and Boosts Heterologous Immunity of hACE2 Transgenic Mice to SARS-CoV-2 Infection

**DOI:** 10.3390/vaccines11111732

**Published:** 2023-11-20

**Authors:** Momtchilo Russo, Maria Cássia Mendes-Corrêa, Bruna B. Lins, Victor Kersten, Paulo C. A. Pernambuco Filho, Toni Ricardo Martins, Tânia Regina Tozetto-Mendoza, Lucy Santos Vilas Boas, Brisa Moreira Gomes, Livia Mendonça Munhoz Dati, Amaro Nunes Duarte-Neto, Gustavo Roncoli Reigado, Ana Beatriz T. Frederico, Danielle R. de A. de Brito e Cunha, Anderson Vicente de Paula, José Igor G. da Silva, Carlos F. Moreira Vasconcelos, Felipe S. Chambergo, Viviane Abreu Nunes, Ana Paula Dinis Ano Bom, Leda R. Castilho, Rodrigo A. P. Martins, Mario Hiroyuki Hirata, Luciana Mirotti

**Affiliations:** 1 Department of Immunology, Institute of Biomedical Science, University of São Paulo (ICB-USP), São Paulo 05508-000, Brazil; 2 Laboratório de Virologia (LIM52), Instituto de Medicina Tropical de São Paulo, Faculdade de Medicina da Universidade de São Paulo (FM-USP), São Paulo 05403-000, Brazil; maria.cassia@hc.fm.usp.br (M.C.M.-C.); toni.martins@ufam.edu.br (T.R.M.);; 3 Faculdade de Ciências Farmacêuticas, Universidade Federal do Amazonas (UFAM), Manaus 69080-900, Brazil; 4 Departamento de Analises Clinicas e Toxicologicas, Faculdade de Ciências Farmacêuticas da Universidade de Sao Paulo (FCF-USP), São Paulo 05508-000, Brazilmhhirata@usp.br (M.H.H.); 5 Departamento de Patologia, Faculdade de Medicina da Universidade de São Paulo (FM-USP), São Paulo 05403-000, Brazil; 6 Laboratório de Biotecnologia, Escola de Artes, Ciências e Humanidades, Universidade de São Paulo (EACH-USP), São Paulo 03828-000, Brazilfscha@usp.br (F.S.C.); vanunes@ib.usp.br (V.A.N.); 7 Immunological Technology Laboratory, Institute of Immunobiological Technology (Bio-Manguinhos), Oswaldo Cruz Foundation (Fiocruz), Rio de Janeiro 21040-900, Braziladinis@bio.fiocruz.br (A.P.D.A.B.); 8 Programa de Biologia Celular e do Desenvolvimento, Instituto de Ciências Biomédicas, Universidade Federal do Rio de Janeiro (UFRJ), Rio de Janeiro 21941-902, Brazilrodrigo.martins@icb.ufrj.br (R.A.P.M.); 9 Cell Culture Engineering Laboratory, COPPE, Federal University of Rio de Janeiro (UFRJ), Rio de Janeiro 21941-598, Brazil; leda@peq.coppe.ufrj.br; 10 Institute of Science and Technology in Biomodels (ICTB), Oswaldo Cruz Foundation (Fiocruz), Rio de Janeiro 21040-900, Brazil

**Keywords:** SARS-CoV-2, vaccine, hACE2 transgenic mice, intranasal route, spike protein, cationic liposome, CpG-ODNs, heterologous immunity

## Abstract

Mucosal vaccination appears to be suitable to protect against SARS-CoV-2 infection. In this study, we tested an intranasal mucosal vaccine candidate for COVID-19 that consisted of a cationic liposome containing a trimeric SARS-CoV-2 spike protein and CpG-ODNs, a Toll-like receptor 9 agonist, as an adjuvant. In vitro and in vivo experiments indicated the absence of toxicity following the intranasal administration of this vaccine formulation. First, we found that subcutaneous or intranasal vaccination protected hACE-2 transgenic mice from infection with the wild-type (Wuhan) SARS-CoV-2 strain, as shown by weight loss and mortality indicators. However, when compared with subcutaneous administration, the intranasal route was more effective in the pulmonary clearance of the virus and induced higher neutralizing antibodies and anti-S IgA titers. In addition, the intranasal vaccination afforded protection against gamma, delta, and omicron virus variants of concern. Furthermore, the intranasal vaccine formulation was superior to intramuscular vaccination with a recombinant, replication-deficient chimpanzee adenovirus vector encoding the SARS-CoV-2 spike glycoprotein (Oxford/AstraZeneca) in terms of virus lung clearance and production of neutralizing antibodies in serum and bronchial alveolar lavage (BAL). Finally, the intranasal liposomal formulation boosted heterologous immunity induced by previous intramuscular vaccination with the Oxford/AstraZeneca vaccine, which was more robust than homologous immunity.

## 1. Introduction

Infection by the severe acute respiratory syndrome coronavirus 2 (SARS-CoV-2) has emerged as one of the major public health problems since 2019 due to the global spread of coronavirus disease (COVID-19) [1]. Paralleling the outbreak of the COVID-19 pandemic was the extraordinary innovation and unprecedented development of highly effective vaccines [2]. For example, all vaccines approved by the European Medical Agency were highly efficacious against severe COVID-19 infection [3]. Indeed, in Brazil, the first model city vaccination program was performed with the CoronaVac vaccine (Sinovac Biotech) in the town of Serrana, São Paulo State, and the result of the efficient immunization campaign was a reduced death toll rate and related COVID-19 morbidity when compared with the rest of Brazil, where the immunization rates were not as high [4]. However, systemic humoral immunity induced by vaccination wanes over time, as revealed by declining neutralizing antibody titers. The intramuscular vaccination, although safe and effective at inducing protective immunity, might fail to induce optimal mucosal immunity in the airways, thus facilitating virus transmission [5]. The emergence of viral variants of concern (VOC) has further complicated the pandemic. To address these challenges, the World Health Organization has recommended vaccine booster shots to enhance immunity [6]. It is anticipated that a mucosal vaccine might be advantageous in this scenario due to its potential to prevent infection and transmission and may be more effective against VOCs as they can induce local immune responses at the sites of viral entry. In this regard, the oral vaccine against poliovirus (the Sabin vaccine) was the first vaccine to prove the concept of mucosal immunity; in contrast to the Salk intramuscular vaccine, the Sabin vaccine prevented the disease as well as its transmission by IgA neutralizing antibodies [7]. Consistently, in COVID-19, IgA antibodies dominated the early SARS-CoV-2-spexcific humoral responses, contributing to virus neutralization to a greater extent than IgG antibodies [8]. Pre-clinical studies in mice confirmed the superior protective immunity to SARS-CoV-2 achieved by intranasal adenovirus-vectored vaccines [9] or intranasal trivalent next-generation COVID-19 vaccines [10]. These reports are in line with previous work showing that mice infected with SARS-CoV sensitized with an adenovirus-vectored vaccine administered by intranasal but not intramuscular routes controlled SARS-CoV replication in the lungs [11]. Besides adaptive humoral immunity, cellular immunity has also been shown to play a role in the control of COVID-19 [12]. Indeed, in murine models of COVID-19, both humoral and cellular adaptive immunity contribute to viral clearance, although the protection from infection appears to be largely mediated by the antibody response [13]. Altogether, vaccines against SARS-CoV-2 might need to be updated periodically and administered preferentially by the mucosal route to avoid loss of clinical efficacy and prevent transmissibility. In addition, given the persistence of the COVID-19 pandemic worldwide, it is highly appropriate that vaccine formulations be versatile and adaptable to different VOCs, stable, and easily manufactured in different countries and under dissimilar conditions.

**Figure 1:**
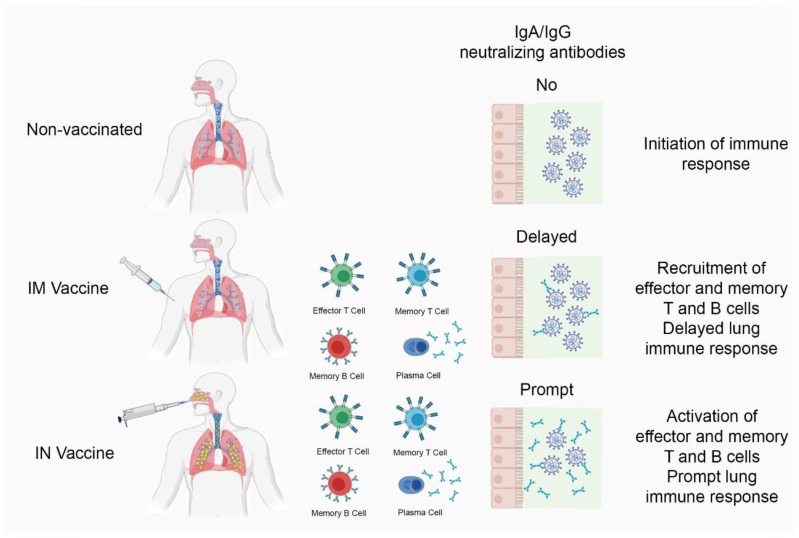
Graph illustration of mechanisms described in the introduction.

Here, we have used a vaccine platform consisting of a cationic liposome containing a recombinant trimeric SARS-CoV-2 spike protein to induce robust T-follicular helper cell and humoral responses [14], adjuvanted with CpG oligonucleotides to boost mucosal IgA antibody production [15] and Th1 cellular immunity, in a transgenic mouse model (K18-hACE2) of COVID-19 [16], aiming to obtain humoral and cellular effector and memory immune responses as depicted in the graphic illustration (Figure 1). The study results show that our candidate COVID-19 vaccine is not toxic and that its intranasal administration induces superior immunity compared with the subcutaneous or intramuscular routes. Also, the intranasal vaccine afforded protection against gamma, delta, and Omicron virus variants. In addition, the intranasal liposomal formulation boosted heterologous immunity, which was better than the homologous immunity induced by previous vaccination with a recombinant, replication-deficient chimpanzee adenovirus vector encoding the SARS-CoV-2 spike glycoprotein (Oxford/AstraZeneca vaccine).

## 2. Material and Methods

### 2.1. Vaccine Formulation

The vaccine formulation contained as antigen the trimeric spike protein of SARS-CoV-2 stabilized in the prefusion conformation, which was provided by the Cell Culture Engineering Laboratory of COPPE/UFRJ by means of serum-free cultivation of stably transfected HEK293 cells and purified by affinity chromatography, as described by Alvim et al. [17]. In all experiments, the spike protein corresponded to the Wuhan strain (1208 aminoacids that form the spike ectodomain), as proposed by Wrapp et al. [18], except for the toxicity study, where the Wuhan aminoacid sequence was slightly altered by one mutation (D614G). The adjuvant was a Class C CpG oligonucleotide (Human/Murine TLR9 ligand, ODN 2395, Invivogen, San Diego, CA, USA, or Exxtend, Campinas, Brazil). Each 30 μL vaccine dose used in mice studies contained 5 μg of spike protein and 10 μg of CpG, entrapped in DOTAP (ROCHE DOTAP Liposomal Transfection Reagent N-[1-(23-Dioleoyloxy) propyl]-NNN-trimethylammonium methyl-sulfate code:11202375001, Basel, Switzerland).

### 2.2. Physicochemical Characterization

Size and polydispersity index (PDI) measurements of the vaccine formulation were performed using the Zetasizer Nano Ultra equipment (Malvern, UK) with a polystyrene cuvette at a backscatter angle (173°). To analyze the surface charge of the particle, specific cuvettes were used to measure the zeta potential. Vaccine formulations were diluted to a volume of 1 mL before measurement. Results were measured in three replicates, which are reported as mean ± standard deviation. To evaluate particle size, we used average hydrodynamic size weighted by particle intensity analyzed by dynamic light scattering (DLS) methodology.

### 2.3. Toxicity of Vaccine Formulation

In vivo toxicity: Female (n = 15) and male (n = 15) CD1 mice were intranasally vaccinated on days 0 and 14, according to the vaccination protocol described below (Vaccination, COVID-19 animal model). Blood samples were collected on days 0 (before vaccination), 2, 16, and 29. Hemogram, albumin/globulin rate, aspartate aminotransferase (AST), alanine transaminase (ALT), alkaline phosphatase (ALP), total bilirubin, urea, creatinine, calcium, phosphorus, total proteins, albumin, glucose, total cholesterol and triglycerides, and alpha-1-glycoprotein were evaluated. Organs were weighted, macro- and microscopically evaluated at days 16 (n = 20) and 29 (n = 10): aorta, spleen, urinary bladder, brain, nasal cavity, tongue, heart, esophagus, stomach, liver, adrenal, mammary, pituitary, and mandibular salivary glands, thyroid and parathyroid, small and large intestines, mesenteric lymph node, cervical lymph nodes (submandibular, near the site of administration), thymus, spinal cord, bone marrow, skeletal muscle, sciatic nerve, femur, ovaries, pancreas, skin, prostate, lungs, kidneys, testicles, epididymis, seminal vesicle, trachea, uterus, cervix, vagina, eyes, optic nerve. Body weight, food consumption, clinical score, morbidity, and mortality were evaluated at least once a day. Body temperature was measured before, 3 h, and 24 h after vaccination. Animal welfare guidelines of CIEnP were followed for all animal procedures under National Brazilian Legislation 11.794 Law, which complies with the commonly accepted 3Rs: reduction, replacement, and refinement (Ethic Protocol CEUA 308/00).

In vitro toxicity: Vaccine cytotoxicity was evaluated according to [19]. Briefly, 3T3 fibroblasts, CALU-3 lung cells, primary human fibroblasts (HLF), and rat lung cells (pneumocytes) were added to flat-bottom 96-well plates (Corning, Salt Lake City, UT, USA) at a concentration of 8000 cells per well. Incubation was performed for 48 h in a humid atmosphere with 5% CO_2_ at 37 °C. After this period, the vaccine formulation diluted in PBS pH 7.2 was added (0.1, 1.0, or 10 µL) in the final volume of 100 µL for 24 or 48 h. The colorimetric method of resazurin (7-hydroxy-3H-phenoxazine-3-one 10-oxide) was used to evaluate cell viability based on the intracellular reduction of resazurin to resorufin by viable and metabolically active cells [20]. The plates were kept with the resazurin solution (0.02 mg/mL) (Sigma-Aldrich, Waltham, MA, USA—cod. M2003) in DMEM without phenol for 2 h at 37 °C. The absorbance of the samples was measured at 570 and 600 nm in a Synergy HT plate reader (Biotek, Winooski, VT, USA) using the Gen Version 5 ™ software (BioTek, Winooski, VT, USA). The tests were performed in sextuplicate, and the results were expressed as the mean ± standard error of at least two different experiments. Cells incubated with PBS were used as negative controls, while cells incubated in 10% dimethyl sulfoxide (DMSO) were used as positive controls for cell death.

### 2.4. COVID-19 Animal Model

Animals: Transgenic C57BL/6 mice (K18-hACE2) expressing the human hACE2 receptor, lineage B6.Cg-Tg(K18-ACE2)2Prlmn/J HEMI Homozygous for Tg(K18-ACE2)2 Prlmn [21] was purchased from Jackson Laboratories and bred at ICTB, Rio de Janeiro, Brazil. Mice were kept in a Specific Pathogen-Free (SPF) Biosafety Level (BSL)-2 animal house and transferred to a BSL-3 environment 2 days before virus infection. Food and water were provided ad libitum. Environmental enrichment was provided in the BSL-2 animal house. Mice were treated according to animal welfare guidelines of ICB—USP (Ethic Protocol 4344010720) under National Legislation 11.794 Law.

Vaccination: Vaccinated animals were injected with (i) the vaccine candidate intranasally (i.n.) in a total volume of 30 μL (15 μL per nostril) or subcutaneously (s.c.), administered in the dorsal neck region (total of 100 μL, prepared by mixing 30 μL of vaccine formulation with 70 μL of sterile saline), or (ii) intramuscularly (i.m.) in the thigh muscles of the hind limb (total of 70 μL, 35 μL per leg) with the Oxford/AstraZeneca (AZ) COVID-19 vaccine (produced at FIOCRUZ/Bio-Manguinhos, Rio de Janeiro, Brazil). Animals were vaccinated on days 0 and 7, and for some experiments, a vaccination booster was administered on day 14. Non-vaccinated mice received only phosphate-buffered saline (PBS). Details of the protocols performed in each experiment are indicated in the figures’ diagrams. Mice were anesthetized intra-peritoneally with ketamine (50 mg/kg, Syntec, São Paulo, Brazil) and xylazine (20 mg/kg, Syntec, São Paulo, Brazil) before each injection.

Virus infection: Four different strains of SARS-Cov-2 were used: Wuhan (wild type).

EPI_ISL_1557222, strain B.1.1.28 obtained from nasopharyngeal swab taken from an infected patient from São Paulo, Brazil, in April 2020; Gamma, EPI_ISL_1060902, strain P.1 was obtained from a nasopharyngeal specimen of a patient from Amazonas, Brazil, in December 2020 (this strain was previously classified as belonging to the P.1 lineage by virus genome sequencing); Delta, EPI_ISL_2938096, strain B.1.617.2, Instituto Butantan, and Ômicron (EPI_ISL_6901961—strain B.1.1.529, Instituto de Ciências Biomédicas, University of São Paulo. K18-hACE2 C57Bl/6J mice were anesthetized as described for vaccinations and were inoculated i.n. with 10ˆ5 TCID50 of SARS-CoV-2 per mouse, in a total volume of 30 μL (15 μL per nostril). Infected mice were kept in Biosafety Level 3 (BSL-3) environment for a maximum of 7 days. Body weight and clinical score were recorded daily, and euthanasia was performed in mice reaching 20% of weight loss or showing signals of suffering, such as decreased activity, piloerection, un-groomed appearance, abnormal stance with ataxia, changes in eye brightness, or change in respiratory pattern. Euthanasia was performed intra-peritoneally with lethal doses of inhaled isoflurane (Cristalia, Itapira, Brazil). Mice were treated according to animal welfare guidelines of FCF-USP (Ethic Protocol CEUA 621) under National Legislation 11.794 Law.

### 2.5. Nucleic Acid Extraction and RT-qPCR (Quantitative Real-Time PCR Based on Reverse Transcriptase) Assay for SARS-CoV-2

Lung lobe tissue samples were macerated mechanically, and simultaneously, viral inactivation and digestion were performed in lysis buffer (containing guanidinium isothiocyanate + proteinases K), according to the manufacturer’s instructions for the Extract kit fast DNA and RNA viral reagents (Loccus^®^, Cotia, São Paulo, Brazil). The specimens underwent nucleic acid extraction and purification by using the automatized extractor EXTRACTA 32 with magnetic beads (Loccus^®^, Cotia, São Paulo, Brazil), according to the manufacturer’s instructions. For in house SARS-CoV-2 quantification by RT-qPCR, specific primers and probes for the E-gene of SARS-CoV2 were synthesized as described previously [22,23]. Standard curves were generated for the quantitative RT-qPCR for E-gene SARS-CoV-2 with known amounts of the synthetic oligos as previously reported [24] by using TaqMan™ Fast Virus 1-Step Master Mix system (Thermo Fisher Scientific^®^, Austin, TX, USA). The data were analyzed using QuantStudio Design & Analysis Software v.1.4.1. The viral load was expressed as the log_10_ number of viral copies per ng of RNA. Samples were handled according to laboratory biosafety guidelines.

### 2.6. RNA Isolation, cDNA Synthesis, and RT-qPCR for Cytokines

Olfactory bulb and hippocampus were dissected in PBS 1x, incubated in RNA later overnight at 4 °C (Sigma Aldrich, cat# R0901, St Louis, MO, USA), and kept at −80 °C. Tissues were lysed using QIAzol reagent (Qiagen, cat# 79306, Germantown, MD, EUA) and the RNeasy Plus Kit (Qiagen, cat# 74134, Germantown, MD, USA) was used for genomic DNA elimination and RNA isolation. RNA integrity was confirmed by electrophoresis on a 1% agarose gel under denaturing conditions. cDNA was synthesized using the High-Capacity RNA-to-cDNA Kit (Thermo Fisher Scientific, cat# 4388950, Waltham, MA, USA), according to the manufacturer’s instructions. RT-qPCR reactions were performed using SYBR green amplifications (PowerTrack SYBR Green Master Mix, Thermo Fisher Scientific, cat# A46109, Waltham, MA, USA). Reactions consisted of 10 μL of SYBR mix, 2 μL of cDNA, 1 μL (10 μM) of each primer, and 6 μL of UltraPure water (Thermo Fisher Scientific, cat# AM9932, Waltham, MA, USA). The cycling conditions were: 95 °C for 20 s, 40 cycles of 95 °C for 1 s and 60 °C for 20 s. Targets and primer sequences are listed below (Table 1). Relative gene expression was determined by applying the 2^-ΔΔCt^ method, using β-Actin expression for normalization, and comparing treated mice with non-infected controls.

### 2.7. Virus Neutralization Test (VNT)

The Cytopathic Effect (CPE)-based Virus Neutralization Test (VNT) was adapted from Nurtop et al. [25] and applied as previously described [26,27,28]. The VNT was performed with SARS-CoV-2 Wuhan (wild type) EPI_ISL_1557222 (described above in the virus infection section) in 96-well microtiter plates containing 5 × 10^4^ Vero cells/mL. These cells were seeded in a 96-well microtiter plate and allowed to grow for 24 h prior to infection. Serum and BAL to be tested were heat-inactivated for 30 min at 56 °C. Then, 110 μL of two-fold serially diluted sera (from 1:20 to 1:2560) were added to the mixed vol/vol with 10^3 TCID50/mL of SARS-CoV-2 and incubated at 37 °C for 1 h for virus neutralization. The sera-virus and BAL-virus mixture was transferred onto the confluent Vero cell monolayer and incubated for 72 h. Cultures at 37 °C and 5% CO_2_ were observed daily for a CPE. After 72 h, the plates were analyzed by light microscopy (Nikkon, Tokyo, Japan), distinguishing the presence/absence of CPE-VNT. To verify the initial observations, 72 h later, the monolayers were fixed and stained with Naphthol Blue Black (Sigma-Aldrich Co., Deisenhofen, Germany) dissolved in sodium acetate-acetic acid for 30 min. Dilutions of serum associated with CPE were considered a negative result. The absence of CPE or complete neutralization of SARS-CoV2 inoculum was considered a positive result. For each reaction, virus diluted in DMEM with 2.5% FBS was used as positive control, while DMEM with 2.5% FBS without added virus served as a negative control. As additional controls, a serum specimen taken from a patient with a SARS-CoV-2 infection was used as a positive control, and a sample from a patient without neutralizing antibodies, with known VNT results, was used as negative control. The antibody titer was calculated as the highest dilution where CPE was completely inhibited. Titers ≥ 1:20 were reported as positive. Virus isolation and VNT were performed in a Biosafety Level (BSL)-3 laboratory.

### 2.8. Enzyme-Linked Immunosorbent Assay (ELISA) for Antibodies

S-Protein-specific IgA was determined by adding serum or BAL (broncho-alveolar lavage) samples at multiple dilutions to 96-well plates previously coated with spike protein provided by the Cell Culture Engineering Laboratory of COPPE/UFRJ, followed by incubation with a goat anti-mouse IgA antibody conjugated to HRP (cat#1040-05, Southern Biotech, Birmingham, AL, USA). Purified mouse IgA (cat#1040-01, Southern Biotech, Birmingham, AL, USA) was used as standard. Serum assays were performed in 96-well plates (cat #442404, Thermo Fisher, Waltham, MA, USA) and BAL assays in 96-well plates (cat#3690, Corning, Corning, NY, USA).

### 2.9. Statistical Analysis

The results were expressed as the mean ± standard error (SE) or standard deviation (SD), as indicated in the figures. Statistically significant differences were determined using unpaired t-tests or ANOVA tests, followed by the Bonferroni post-test, as applicable and according to test requirements. A *p-*value < 0.05 was considered statistically significant (* *p* < 0.05, ** *p* < 0.01, *** *p* < 0.001). GraphPad Prism 8 (GraphPad Software, La Jolla, CA, USA) was used for statistical analysis and graph generation. The experiments were not randomized, and the investigators were not blinded to allocation during experiments and outcome assessment.

## 3. Results

### 3.1. Toxicity of Nasal Vaccine Formulation

The toxicity of the nasal vaccine formulation was tested using in vivo and in vitro models. The in vivo toxicity study is a pre-clinical test required by the Brazilian Sanitary Regulatory Agency (ANVISA) for approval of vaccine clinical trials in Brazil. Therefore, the tests were performed under good laboratory practices (GLP) by CIEnP (Center of Innovation and Pre-clinical Studies), which is accredited by the Brazilian Accreditation Body (INMETRO). The spike protein used in the in vivo toxicology study contained one single mutation as compared with the Wuhan sequence (a G instead of a D in residue 614). The D614G mutation has become prevalent in all circulating SARS-CoV-2 strains worldwide since Q2/2020. Since the spike protein ectodomain used as an antigen contains 1280 aminoacids, the single D614G mutation means a 99.92% homology in terms of aminoacid sequence. No differences in toxicology or immunogenicity are expected between Wuhan and the D614G mutant spike, due to the >99.9% similarity.

The D614G protein was used for this test because this is the protein that was produced under Good Manufacturing Practices (GMP) conditions by the Federal University of Rio de Janeiro for use in clinical trials, and it is recommended that the toxicology study be carried out with the same antigen lot as the clinical trial. The parameters evaluated included clinical score, morbidity, mortality, body temperature, body weight, food consumption, hemogram, biochemical blood parameters, and histopathology of several organs, as detailed in M&M. No toxicity was recorded after intranasal vaccine administration Table S1).

**Figure 2:**
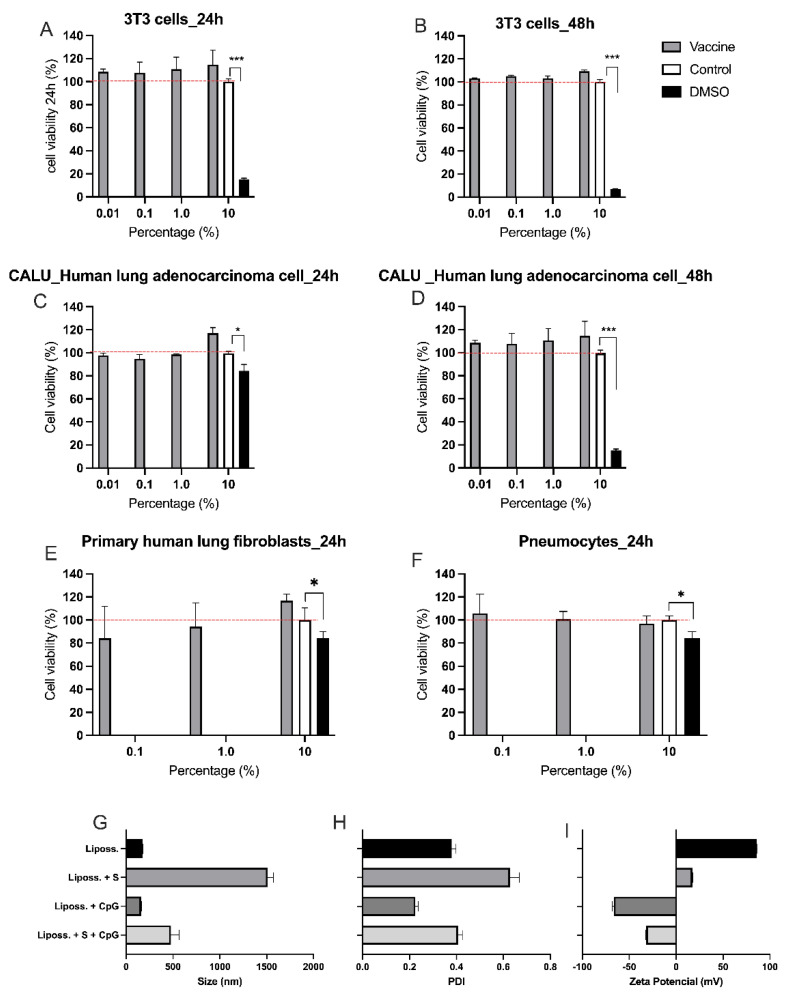
Cytotoxicity assays for vaccine formulation. (**A**–**F**) Incubation of different cell types with nasal vaccine formulation (grey bars), PBS—negative controls (white bars), or DMSO—positive controls (black bars). Axis X indicates the percentage of vaccine formulation, PBS, or DMSO present in the wells. (**A**) Fibroblasts 3T3 incubated for 24 h; (**B**) Fibroblasts 3T3 incubated for 48 h; (**C**) CALU-3—human lung adenocarcinoma cells—incubated for 24 h; (**D**) CALU-3—human lung adenocarcinoma cells—incubated for 48 h; (**E**) Human lung fibroblasts incubated for 24 h; (**F**) Rat pneumocytes were incubated with vaccine formulation for 24 h. Cytotoxicity assays were performed by resazurin method. Cultures incubated with 10% DMSO were positive controls for cell death. Evaluation of the physicochemical characteristics of different formulations using the dynamic light scattering (DLS) technique (**G**) Hydrodynamic size (nm); (**H**) Charge (zeta potential—mV); (**I**) Polydispersity index (PDI). (**A**–**F**) The data are presented as the mean ± SE of four individual experiments, in sextuplicates. (**G**–**I**) The data are presented as the mean ± SE of three individual experiments, in triplicate. * *p* < 0.05, *** *p* < 0.001.

For in vitro toxicity tests, four different cell types were used. Firstly, 3T3 cells, a standardized cell lineage for cytotoxicity assays, served to verify that the vaccine formulation was not cytotoxic at any of the concentrations and periods of incubation (Figure 2A,B). The viability of CALU-3 cells, a human lung cancer cell line used as a respiratory model in preclinical applications [29], was then assessed in the presence or absence of the vaccine formulation, showing that the formulation was not cytotoxic (Figure 2C,D). Finally, human lung fibroblasts and primary rat pneumocytes remained viable after 24 h of incubation with the vaccine formulation, even at the highest concentrations tested (Figure 2E,F).

The physicochemical characterization of the vaccine formulation, evaluated at different stages, demonstrated that, concerning size, there is a significant increase in the formulation when only the S protein is added to the DOTAP liposome. In this context, when CpG is subsequently added to this mixture, the size is reduced. When we compare the DOTAP liposome with and without the addition of CpG, without protein, we do not observe a significant modification in size (Figure 2G). The same effect is observed in the polydispersity, with a decrease in the polydispersity index (PDI) upon adding CpG compared with samples without the adjuvant, suggesting that the addition of the adjuvant is beneficial for the formulation in terms of physicochemical parameters (Figure 2H). Regarding the charge of the vaccine formulation, we found that without the adjuvant, the formulation has a positive charge, and only with its presence does it become negative (Figure 2I).

### 3.2. The Effect of Intranasal Versus Subcutaneous Vaccine Administration

**Figure 3:**
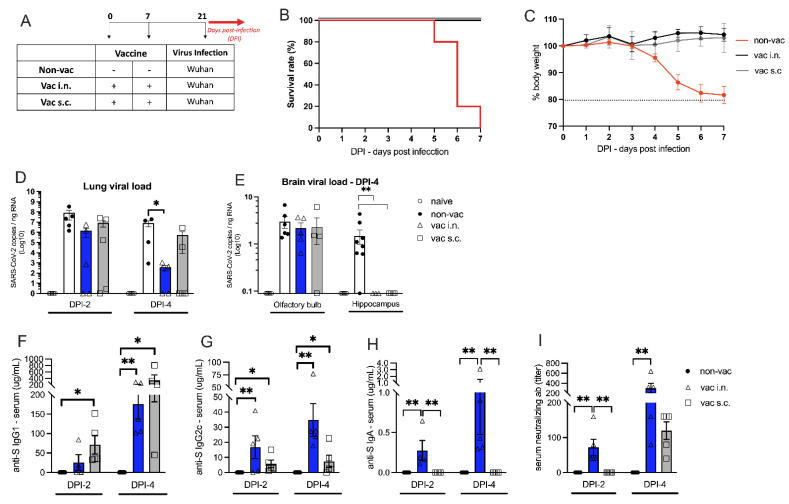
Intranasal versus subcutaneous vaccinations. (**A**) Protocol design: K18-hACE2 mice were vaccinated via intra-nasal (vac i.n.) or via sub-cutaneous (vac s.c.) with the nasal vaccine formulation on days 0 and 7. Non-vaccinated mice (non-vac) received only PBS. All mice were infected intra-nasally with SARS-CoV-2 (Wuhan strain) 21 days after the first dose; (**B**) Survival rate following the virus infection (DPI-days after infection) represented by a Kaplan-Meier survival curve; (**C**) Body weight during the course of infection plotted as percent (100% representing the body weight on DPI-0 before the virus infection). (**B**,**C**) Red line represents non-vaccinated mice, gray line represents subcutaneously vaccinated mice and black line represents mice vaccinated intranasally; (**D**) RNA was isolated from the lungs and SARS-CoV-2 was quantitated by RT-qPCR measuring E-gene log copies number per ng RNA at two and four days after viral infection, DPI-2 and DPI-4 respectively (**E**) RNA was isolated from distinct brain regions (olfactory bulb and hippocampus) and SARS-CoV-2 was quantitated by RT-qPCR measuring E-gene log copies number per ng RNA four days after viral infection (DPI-4); (**F**) Concentration of spike protein (S)-specific IgG1 in serum measured by ELISA; (**G**) Concentration of S-specific IgG2c in serum measured by ELISA; (**H**) Concentration of S-specific IgA in serum measured by ELISA; (**I**) Titers of SARS-CoV-2 neutralizing antibodies in serum measured by VNT, two and four days after virus infection (DPI-2 an DPI-4). N = 5 per group. Data represent experiments repeated at least three times. * *p* < 0.05, ** *p* < 0.01.

Transgenic K18-hACE2 mice received the vaccine formulation intranasally (i.n.) or subcutaneously (s.c.) on days 0 and 7. Two weeks after the second dose, vaccinated (i.n. or s.c.) or non-vaccinated (non-vac) animals were challenged i.n. with the wild type (Wuhan) SARS-CoV-2 virus (Figure 3A). All infected non-vaccinated mice died after 5 to 7 days post-infection (DPI 5-7), while no mortality was observed in the i.n. or s.c. vaccinated groups (Figure 3B). Non-vaccinated mice started to lose weight 4 days after receiving SARS-CoV-2, reaching 20% of weight loss at DPI-7 (Figure 3C). According to mortality data, both i.n. and s.c. vaccinated groups maintained stable body weight for 7 consecutive days after SARS-CoV-2 infection. These results indicate that the i.n. or s.c. vaccination was equally protective against the SARS-CoV-2 infection.

The viral load in the lung (Figure 3D) and in the brain (Figure 3E) was measured by RT-qPCR on different days after viral infection. Two days after viral infection (DPI-2), 40% of the vaccinated animals (n = 2) (i.n. and s.c.) had no virus detected in the lung tissue (Figure 3D). However, at DPI-2, there was no statistically significant difference between the vaccinated and non-vaccinated groups. At DPI-4, there was a significant decrease in the lung viral load only in the i.n. vaccinated group when compared with non-vaccinated mice (Figure 3D). Moreover, at DPI-6, in the i.n. vaccinated group, SARS-CoV-2 mRNA could not be detected in the lung, while in non-vaccinated animals, a high viral load was detected (Figure S1A).

Next, we determined the viral load in the proximal and distal sites of the nervous system (i.e., closer to and further away from the site of infection, respectively). Interestingly, all vaccinated and non-vaccinated groups were equally positive for the virus in the olfactory bulb (proximal site), but only non-vaccinated animals had the virus in distal areas such as the hippocampus (Figure 3E). As expected, in the lung and brain, SARS-CoV-2 was not detected in naive animals that did not receive the virus (Figure 3D,E). These results indicate that both vaccination routes are equally effective in preventing the virus from spreading to the hippocampus, but the i.n. vaccination was more effective than the s.c. vaccination in clearing the virus from the lung tissue. We further assessed brain inflammation to test whether the i.n. vaccination could prevent neuroinflammation. Following infection, the proinflammatory cytokines IL-1β, TNFα, and IL-6 were upregulated in the olfactory bulb and hippocampus at DPI-4. The i.n. vaccination prevented the upregulation of these cytokines in both brain regions. (Figure S1D,E).

We also measured the levels of anti-spike protein (S) antibodies in the serum of vaccinated and non-vaccinated K18-hACE2 mice. The levels of anti-S IgG1 at DPI-2 were significantly higher in the s.c.-vaccinated mice when compared with non-vaccinated mice (Figure 3F). Although mice vaccinated by the i.n. route had detectable anti-S IgG1 in the serum, there was no statistically significant difference compared with the non-vaccinated animals. However, at DPI-4, mice in both vaccinated groups presented significantly higher levels of anti-S IgG1 than the animals from the non-vaccinated group (Figure 3F). The levels of IgG1 antibodies were generally higher at DPI-4 when compared with DPI-2 (Figure 3F). The levels of anti-S IgG2c at DPI-2 and DPI-4 were significantly higher in both groups of vaccinated animals (s.c. and i.n.) when compared with the non-vaccinated group (Figure 3G), with antibody levels generally higher at DPI-4 when compared with DPI-2 (Figure 3G). Importantly, serum anti-S IgA was only found in i.n. vaccinated mice that showed significantly higher IgA titers than non-vaccinated or s.c. vaccinated animals (Figure 3H). Similar anti-S IgA results were found in the bronchoalveolar lavage (BAL) (Figure S1B). As expected, no anti-S immunoglobulins were found in non-vaccinated animals.

For all antibodies measured, the levels were higher at DPI-4 than at DPI-2, indicating that antibody production increased after viral infection. Altogether, these results indicate that the vaccine formulation was effective in inducing the production of anti-S antibodies and that the i.n. route induced higher levels of specific IgG2c and IgA antibodies when compared with the s.c. administration.

Finally, the titers of SARS-CoV-2 neutralizing antibodies in the serum of vaccinated and non-vaccinated mice were measured by VNT (Figure 3I). At DPI-2, neutralizing antibodies were found only in i.n.-vaccinated animals. At DPI-4, although neutralizing antibodies were detected in the serum of s.c. vaccinated animals, this was not significantly different from non-vaccinated animals. In contrast, the titers of neutralizing antibodies found in i.n.-vaccinated mice were highly increased compared with the non-vaccinated group. The antibody titers were also increased in DPI-4 in relation to DPI-2, confirming the progressive increase in antibody production in i.n.-vaccinated mice after contact with the virus.

In conclusion, both vaccine administration routes were highly effective in protecting mice against SARS-CoV-2 infection, but the i.n. route was more efficient in clearing SARS-CoV-2 from the lung and inducing the production of S-specific IgG2c and IgA antibodies and neutralizing antibodies in the serum than the s.c. route. Therefore, we selected the i.n. vaccination to perform our next experiments.

### 3.3. Intranasal Vaccine Protects against SARS-CoV-2 Variants

**Figure 4:**
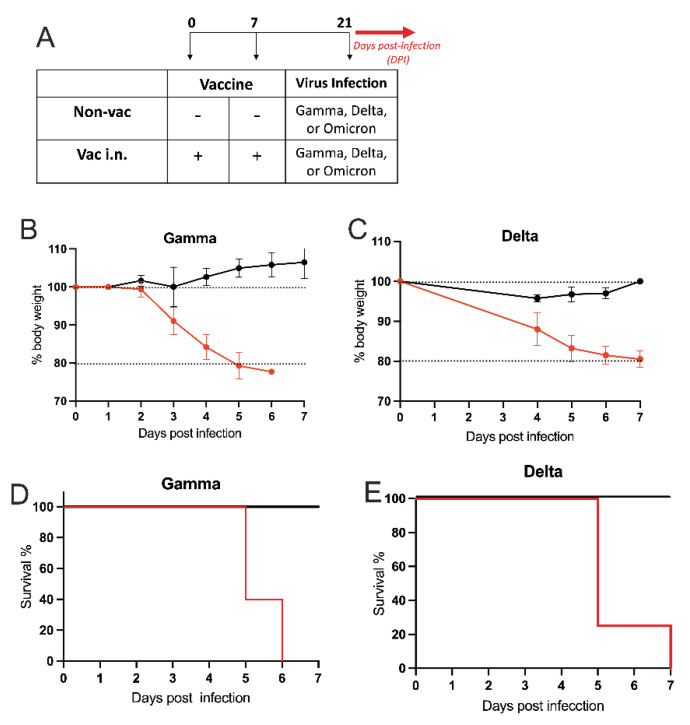
Different SARS-CoV-2 variants. (**A**) Protocol design: K18-hACE2 mice were vaccinated via intra-nasal (vac i.n.) with the nasal vaccine formulation on days 0 and 7. Non-vaccinated mice (non-vac) received PBS. All mice were infected intra-nasally with different SARS-CoV-2 VOC (Gamma or Delta strains) 21 days after the first dose. (**B**,**C**) Body weight during the course of infection plotted as percent (100% representing the body weight on DPI-0 before the virus infection). (**B**) animals infected with Gamma variant of concern (VOC) and (**C**) animals infected with Delta VOC. (**D,E**) Survival rate following the virus infection (DPI—days after infection) is represented by a Kaplan-Meier survival curve: (**D**) animals infected Gamma VOC; (**E**) animals infected Delta VOC. N = 5 per group. Data represent experiments repeated at least three times. Red line: non-vac, black line: vac i.n.

Having established the effectiveness of the i.n. vaccination, we investigated whether the i.n. vaccination could protect mice against infection with different variants of concern (VOC) isolated from patients with COVID-19. For this, transgenic hACE2 mice received i.n. vaccination or PBS on days 0 and 7, and two weeks later, the animals were challenged i.n. with different variants of the active SARS-CoV-2 virus as depicted in Figure 4A. As shown in Figure 4B, mice from the non-vaccinated group infected with the gamma variant started to lose weight 3 days after viral infection, reaching 20% of weight loss at DPI-5. In addition, 60% of mortality was observed in this group of non-vaccinated animals at DPI-5, and by DPI-6, all animals from the group had died (Figure 4D). In contrast, intranasally vaccinated mice did not show weight loss or mortality after the infection with the gamma variant (Figure 4B,D). Similar results were obtained when mice were infected with the delta variant of SARS-CoV-2 (Figure 4C,E). Again, non-vaccinated animals presented significant weight loss and high mortality (80% by DPI-6), while i.n. vaccinated mice did not present weight loss or mortality after the infection with the delta variant. Regarding the Omicron strain, we only measured the viral load in the lungs of vaccinated and non-vaccinated animals since this variant does not induce weight loss or mortality in hACE2 mice [30]. We found that vaccinated mice presented a significantly lower viral load in the lung than non-vaccinated animals at DPI-4 (Figure S1C). We conclude that i.n. vaccination afforded protections against all VOC tested.

### 3.4. Comparison of Intranasal Liposomal Vaccine with the Intramuscular Adenoviral-Vectored SARS-CoV-2 Vaccine from Oxford/AstraZeneca (AZ)

**Figure 5:**
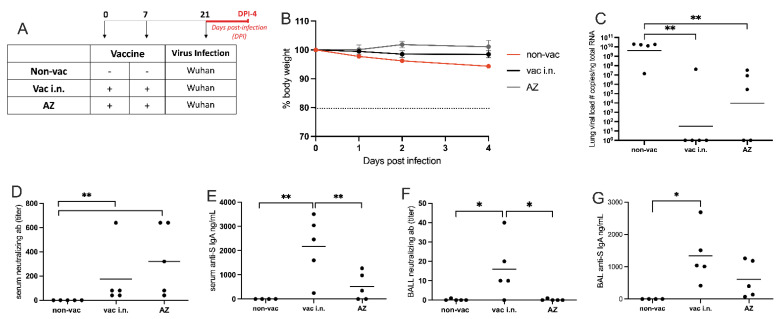
Nasal formulation versus Oxford/AstraZeneca vaccination. (**A**) Protocol design: K18-hACE2 mice were vaccinated on days 0 and 7 with nasal vaccine formulation via intra-nasal (vac i.n.) or vaccinated with Oxford/AstraZeneca vaccine via intramuscular (AZ). Non-vaccinated mice (non-vac) received PBS. All mice were infected intra-nasally with SARS-CoV-2 (Wuhan strain) 21 days following the first dose. All samples were collected four days after infection (DPI-4). (**B**) Body weight during the course of infection plotted as percent change (100% representing the body weight on DPI-0, before the virus infection); (**C**) RNA was isolated from the lungs and SARS-CoV-2 was quantitated by RT-qPCR measuring E-gene log copies number per ng RNA; (**D**) Titers of SARS-CoV-2 neutralizing antibodies in serum measured by VNT; (**E**) Concentration of spike (S)-specific IgA in serum measured by ELISA; (**F**) Titers of SARS-CoV-2 neutralizing antibodies in BAL (broncho-alveolar lavage) measured by VNT; (**G**) Concentration of S-specific IgA in BAL measured by ELISA. N = 5 per group; * *p* < 0.05, ** *p* < 0.01. Dot mean non-vac.

In order to compare the effectiveness of our i.n. vaccine with vaccine formulations currently used during the COVID-19 pandemic, we vaccinated transgenic hACE2 mice with our intranasal vaccine formulation or with the adenoviral-vectored SARS-CoV-2 vaccine from Oxford/AstraZeneca (AZ) via the intramuscular (i.m.) route on days 0 and 7. The animals were infected with the WT (Wuhan) strain of SARS-CoV-2 two weeks after the second vaccination. The infected groups were evaluated over a 4-day period after the viral infection, and samples were collected at DPI-4 (Figure 5A). While the body weight of vaccinated animals was stable for 4 days after SARS-CoV-2 infection, the non-vaccinated group showed approximately 5% weight loss at DPI-4 (Figure 5B).

The SARS-CoV-2 viral load in the lung was measured by RT-qPCR, and both vaccinated groups showed significantly lower viral loads in the lung than the non-vaccinated group (Figure 5C). Although the viral load between vaccinated groups did not reach a significant statistical difference, we found that only 20% of the animals that received the i.n. vaccine tested positive for viral mRNA, while 60% of the animals tested positive in the AZ-vaccinated group at DPI-4 (Figure 5C).

We also measured the levels of SARS-CoV-2 neutralizing antibodies in serum and in the BAL by VNT (Figure 5D,F). In the serum, both vaccinated groups induced higher levels of neutralizing antibodies than non-vaccinated animals (Figure 5D). However, in the BAL, only mice that received the intranasal vaccine produced neutralizing antibodies (Figure 5F). Finally, the concentrations of S-specific IgA antibodies were measured in the serum and BAL by ELISA (Figure 5E,G). Mice vaccinated with the i.n. formulation showed significantly higher levels of anti-S IgA in the serum when compared with non-vaccinated or AZ-vaccinated mice (Figure 5E). Also, in the BAL, the IgA levels were significantly higher in intranasally vaccinated animals when compared with AZ- or non-vaccinated groups (Figure 5G).

Altogether, these data indicate that the i.n. vaccination was more effective than the i.m. AZ vaccination to induce neutralizing antibodies in the lung and anti-S antibodies in serum and in the lung.

### 3.5. Intranasal Vaccine Boosts Heterologous Immunity and Is More Effective Than Homologous Oxford/AstraZeneca (AZ) Vaccine Boost

**Figure 6:**
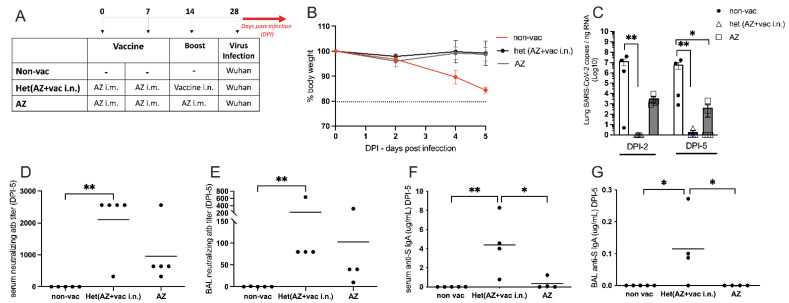
Heterologous vaccination: Booster with nasal formulation versus Oxford/AstraZeneca. (**A**) Protocol design: all K18-hACE2 mice were vaccinated with Oxford/AstraZeneca via intramuscular on days 0 and 7. On 14th day, mice were boosted either with the nasal formulation via intra-nasal Het(AZ+vaci.n) or with Oxford/AstraZeneca via intramuscular (AZ). Non-vaccinated animals (non-vac) received only PBS. All mice were infected intra-nasally with SARS-CoV-2 (Wuhan strain) 28 days after the first dose. (**B**) Body weight during the course of infection plotted as percent change (100% representing the body weight on DPI-0 before the virus infection). (**C**) RNA was isolated from the lungs and SARS-CoV-2 was quantitated by RT-qPCR measuring E-gene log copies number per ng RNA at two (DPI-2) and five (DPI-5) days after viral infection; (**D**–**G**) Samples collected five days after viral infection (DPI-5); (**D**) Titers of SARS-CoV-2 neutralizing antibodies in serum measured by VNT; (**E**) Titers of SARS-CoV-2 neutralizing antibody in BAL (broncho-alveolar lavage) measured by ELISA; (**F**) Concentration of S-specific IgA in serum measured by ELISA; (**G**) Concentration of S-specific IgA in BAL measured by ELISA. N = 5 per group; * *p* < 0.05, ** *p* < 0.01.

Considering that most of the world population has been vaccinated for COVID-19, we were interested in testing the efficiency of our nasal vaccine formulation to boost previous immunity (heterologous immunity) induced by the Oxford/AstraZeneca i.m. vaccination when compared with an AZ i.m. homologous booster. For this, hACE2 transgenic mice received 2 i.m. doses of AZ vaccine on days 0 and 7 and were boosted with the i.n. vaccine formulation or with the i.m. AZ vaccine on day 14. One week after the boost, animals were infected with SARS-CoV-2, as depicted in Figure 6A. We found that the non-vaccinated group lost weight after viral infection, while both vaccinated groups maintained stable body weight after the infection with SARS-CoV-2 (Figure 6B), confirming that vaccination with the AZ formulation protects against infection. Next, we determined the viral load in the lungs 2 days or 5 days after the virus infection. We found that at DPI-2, no virus was detected in mice that received the i.n. vaccine boost, being significantly different from the non-vaccinated group in Figure 6C. Conversely, all animals that received the AZ boost presented virus in the lung at DPI-2, at levels of viral load that were not significantly different from the non-vaccinated group (Figure 6C left). At DPI-5, although AZ-boosted mice had significantly less virus than the non-vaccinated group, 40% of the mice in the AZ-boosted group still had SARS-CoV-2 mRNA present in the lungs (Figure 6C, right). These results indicate that the heterologous i.n. boost is more efficient than the homologous i.m. boost in virus clearance from the lungs. In the same vein, the titers of SARS-CoV-2 neutralizing antibodies in the serum and BAL were significantly higher in animals that received the i.n. boost when compared with the non-vaccinated group (Figure 6D,E). Of note, although neutralizing antibodies were detected in AZ-boosted animals, they were not statistically different from the non-vaccinated group (Figure 6D,E). Finally, animals that received the i.n. boost produced significantly higher levels of anti-S IgA antibodies when compared with the AZ-vaccinated or non-vaccinated groups (Figure 6F,G).

Altogether, these results show that AZ-vaccinated animals that received a heterologous boost with the i.n. vaccine formulation cleared the virus more rapidly and had higher titers of neutralizing antibodies and S-specific IgA in serum and the BAL than mice that received a homologous AZ vaccine boost. Therefore, the heterologous i.n. boost induces more effective anti-SARS-CoV-2 immunity than the homologous AZ boost.

## 4. Discussion

Here we have developed a COVID-19 vaccine formulation using the SARS-CoV-2 trimeric spike protein and CpG type C entrapped in a cationic liposome. We designed this type of formulation to boost both humoral and cellular immunity, based on our previous work with allergen-specific immunotherapy with CpG using the OVA asthma model [31,32]. Also, it was shown that mice immunized with two doses of recombinant virus antigens mixed with cationic adjuvants showed higher serum IgG titers than animals treated with anionic adjuvants [33]. Moreover, lipid nanoparticles enhance the efficacy of mRNA and protein subunit vaccines by inducing robust follicular T-helper cell and humoral responses [14]. Also, our formulation contained CpG type C, which is a potent inducer of IFN-α, a strong B-cell activator in humans and mice [34], and a potent Th1 adjuvant [35].

We found that our vaccine formulation was devoid of toxicity either in cell cultures with different cell types, including human bronchial cells, or in preclinical experiments in CD1 mice. We tested the efficacy of our candidate vaccine in hACE2 transgenic mice infected with the wild-type Wuhan strain of SARS-CoV-2 and compared the results obtained with s.c. versus i.n. administration. Regarding survival and body weight lost, non-vaccinated animals succumbed to infection, while vaccination by both routes prevented viral spread into distal areas of the brain and protected all infected animals. These results are in line with other studies showing the effectiveness of different vaccine formulations administered by different routes [36] and indicate that our vaccine candidate is effective irrespective of the administration route. However, major differences between s.c. and i.n. vaccinations were observed regarding lung viral load, anti-S IgA and IgG2c titers, and levels of neutralizing antibodies in serum. All these parameters of humoral immunity were superior in animals vaccinated by the i.n. route. The enhanced IgA production might reflect the increased antibody neutralizing activity since it was shown that IgA dominates the early neutralizing antibody response to SARS-CoV-2 [8], while the increased IgG2c production might be associated with enhanced Th1 immunity [37]. It was expected that i.n. vaccination would increase IgA production since mucosal delivery of vaccines targets the inductive sites for IgA responses on mucosal surfaces [38,39,40]. In addition, CpG activates the adaptor protein MyD88, which boosts IgA production [15]. In the same vein, CpG also boosts IgG2c production [31]. However, it remains to be determined why i.n. vaccination induces higher titers of IgG2c than s.c. vaccination.

Experimental evidence indicates that vaccine-induced immunity to SARS-CoV-2 infection in hACE2 transgenic mice relies on humoral immunity and/or T cell-mediated immunity [12]. Since our nasal vaccination also afforded protection against infections with VOC that are known to be more resistant to neutralizing antibodies [41], it is likely that our vaccine also induced T cell immunity. In line with this assumption, it was shown that an intranasal COVID-19 vaccine induced respiratory memory T cells and protected K18-hACE mice against SARS-CoV-2 infection [42].

We found that intranasal vaccination was superior to intramuscular vaccination with the Oxford/AstraZeneca vaccine regarding virus clearance from the lung and the production of neutralizing antibodies in serum and BAL. Finally, the intranasal liposomal formulation prevented neuroinflammation and boosted heterologous immunity induced by previous vaccination with the AZ vaccine.

The durability and breadth of our candidate vaccine remain to be determined, but it has been shown previously that nanoparticle-conjugated TLR9 agonists improve the potency, durability, and breadth of COVID-19 vaccines [43].

We conclude that our vaccine formulation is easy to manufacture worldwide. This type of vaccine formulation could be adapted to be used against VOC or different infectious agents that cause pulmonary disease or to boost immunity induced by other vaccines.

## Figures and Tables

**Table 1:** List of primers used for real-time RT-PCR.

Gene	Forward Primer	Reverse Primer
*Actb*	5′-GAA GAT CAT TGC TCC TC-3′	5′-CCT GCT TGC TGA TCC ACA TC-3′
*Il1b*	5′-CAG GCA GGC AGT ATC ACT CA-3′	5′-AGC TCA TAT GGG TCC GAC AG-3′
*Il6*	5′-TAG TCC TTC CTA CCC CAA TTT CC-3′	5′-TTG GTC CTT AGC CAC TCC TTC-3′
*Tnf*	5′-TGT AGC CCA CGT CGT AGC AAA-3′	5′GGC TCA GCC ACT CCA GCT G-3′

## Data Availability

Data are available from the corresponding author upon reasonable request.

## Supplementary Materials

The following supporting information can be downloaded at: https://www.mdpi.com/article/10.3390/vaccines11111732/s1, Figure S1: Additional data regarding Figure 3 and Figure 4; Table S1: In vivo toxicity studies.

## References

[1] Seyed Hosseini E., Riahi Kashani N., Nikzad H., Azadbakht J., Hassani Bafrani H., Haddad Kashani H. (2020). The Novel Coronavirus Disease-2019 (COVID-19): Mechanism of Action, Detection and Recent Therapeutic Strategies. Virology.

[2] Collins F.S., Schwetz T.A., Tabak L.A., Lander E.S. (2021). ARPA-H: Accelerating Biomedical Breakthroughs. Science.

[3] Harder T., Külper-Schiek W., Reda S., Treskova-Schwarzbach M., Koch J., Vygen-Bonnet S., Wichmann O. (2021). Effectiveness of COVID-19 Vaccines against SARS-CoV-2 Infection with the Delta (B.1.617.2) Variant: Second Interim Results of a Living Systematic Review and Meta-Analysis, 1 January to 25 August 2021. Eurosurveillance.

[4] Slavov S.N., de La-Roque D.G.L., da Costa P.N.M., Rodrigues E.S., Santos E.V., Borges J.S., Evaristo M., de Matos Maçonetto J., Marques A.A., Milhomens J. (2022). Dynamics of SARS-CoV-2 Variants of Concern in Vaccination Model City in the State of Sao Paulo, Brazil. Viruses.

[5] Levin E.G., Lustig Y., Cohen C., Fluss R., Indenbaum V., Amit S., Doolman R., Asraf K., Mendelson E., Ziv A. (2021). Waning Immune Humoral Response to BNT162b2 COVID-19 Vaccine over 6 Months. N. Engl. J. Med..

[6] Wang Z., Muecksch F., Muenn F., Cho A., Zong S., Raspe R., Ramos V., Johnson B., Tanfous T.B., Dasilva J. (2022). Humoral Immunity to SARS-CoV-2 Elicited by Combination COVID-19 Vaccination Regimens. J. Exp. Med..

[7] Ogra P.L. (1984). Mucosal Immune Response to Poliovirus Vaccines in Childhood. Rev. Infect. Dis..

[8] Sterlin D., Mathian A., Miyara M., Mohr A., Anna F., Claër L., Quentric P., Fadlallah J., Devilliers H., Ghillani P. (2021). IgA Dominates the Early Neutralizing Antibody Response to SARS-CoV-2. Sci. Transl. Med..

[9] Hassan A.O., Shrihari S., Gorman M.J., Ying B., Yaun D., Raju S., Chen R.E., Dmitriev I.P., Kashentseva E., Adams L.J. (2021). An Intranasal Vaccine Durably Protects against SARS-CoV-2 Variants in Mice. Cell Rep..

[10] Afkhami S., D’Agostino M.R., Zhang A., Stacey H.D., Marzok A., Kang A., Singh R., Bavananthasivam J., Ye G., Luo X. (2022). Respiratory Mucosal Delivery of Next-Generation COVID-19 Vaccine Provides Robust Protection against Both Ancestral and Variant Strains of SARS-CoV-2. Cell.

[11] See R.H., Zakhartchouk A.N., Petric M., Lawrence D.J., Mok C.P.Y., Hogan R.J., Rowe T., Zitzow L.A., Karunakaran K.P., Hitt M.M. (2006). Comparative Evaluation of Two Severe Acute Respiratory Syndrome (SARS) Vaccine Candidates in Mice Challenged with SARS Coronavirus. J. Gen. Virol..

[12] Castro J.T., Azevedo P., Fumagalli M.J., Hojo-Souza N.S., Salazar N., Almeida G.G., Oliveira L.I., Faustino L., Antonelli L.R., Marçal T.G. (2022). Promotion of Neutralizing Antibody-Independent Immunity to Wild-Type and SARS-CoV-2 Variants of Concern Using an RBD-Nucleocapsid Fusion Protein. Nat. Commun..

[13] Israelow B., Mao T., Klein J., Song E., Menasche B., Omer S.B., Iwasaki A. (2021). Adaptive Immune Determinants of Viral Clearance and Protection in Mouse Models of SARS-CoV-2. Sci. Immunol..

[14] Alameh M.G., Tombácz I., Bettini E., Lederer K., Sittplangkoon C., Wilmore J.R., Gaudette B.T., Soliman O.Y., Pine M., Hicks P. (2021). Lipid Nanoparticles Enhance the Efficacy of MRNA and Protein Subunit Vaccines by Inducing Robust T Follicular Helper Cell and Humoral Responses. Immunity.

[15] Suzuki K., Maruya M., Kawamoto S., Sitnik K., Kitamura H., Agace W.W., Fagarasan S. (2010). The Sensing of Environmental Stimuli by Follicular Dendritic Cells Promotes Immunoglobulin A Generation in the Gut. Immunity.

[16] Bao L., Deng W., Huang B., Gao H., Liu J., Ren L., Wei Q., Yu P., Xu Y., Qi F. (2020). The Pathogenicity of SARS-CoV-2 in HACE2 Transgenic Mice. Nature.

[17] Alvim R.G.F., Lima T.M., Rodrigues D.A.S., Marsili F.F., Bozza V.B.T., Higa L.M., Monteiro F.L., Abreu D.P.B., Leitão I.C., Carvalho R.S. (2022). From a Recombinant Key Antigen to an Accurate, Affordable Serological Test: Lessons Learnt from COVID-19 for Future Pandemics. Biochem. Eng. J..

[18] Wrapp D., Wang N., Corbett K.S., Goldsmith J.A., Hsieh C.-L., Abiona O., Graham B.S., Mclellan J.S. (2020). Cryo-EM Structure of the 2019-NCoV Spike in the Prefusion Conformation. Science.

[19] Reigado G.R., Adriani P.P., dos Santos J.F., Freitas B.L., Fernandes M.T.P., Chambergo Alcalde F.S., Leo P., Nunes V.A. (2022). Delivery of Superoxide Dismutase by TAT and Abalone Peptides for the Protection of Skin Cells against Oxidative Stress. Biotechnol. Appl. Biochem..

[20] Präbst K., Engelhardt H., Ringgeler S., Hübner H. (2017). Basic Colorimetric Proliferation Assays: MTT, WST, and Resazurin. Methods in Molecular Biology.

[21] McCray P.B., Pewe L., Wohlford-Lenane C., Hickey M., Manzel L., Shi L., Netland J., Jia H.P., Halabi C., Sigmund C.D. (2007). Lethal Infection of K18- HACE2 Mice Infected with Severe Acute Respiratory Syndrome Coronavirus. J. Virol..

[22] Chu D.K.W., Pan Y., Cheng S.M.S., Hui K.P.Y., Krishnan P., Liu Y., Ng D.Y.M., Wan C.K.C., Yang P., Wang Q. (2020). Molecular Diagnosis of a Novel Coronavirus (2019-NCoV) Causing an Outbreak of Pneumonia. Clin. Chem..

[23] Corman V.M., Landt O., Kaiser M., Molenkamp R., Meijer A., Chu D.K.W., Bleicker T., Brünink S., Schneider J., Schmidt M.L. (2020). Detection of 2019 Novel Coronavirus (2019-NCoV) by Real-Time RT-PCR. Eurosurveillance.

[24] Mendes-Correa M.C., Salomão M.C., Ghilardi F., Tozetto-Mendoza T.R., Santos Villas-Boas L., de Paula A.V., Paiao H.G.O., da Costa A.C., Leal F.E., de Barros Coscelli Ferraz A. (2023). SARS-CoV-2 Detection and Culture in Different Biological Specimens from Immunocompetent and Immunosuppressed COVID-19 Patients Infected with Two Different Viral Strains. Viruses.

[25] Nurtop E., Villarroel P.M.S., Pastorino B., Ninove L., Drexler J.F., Roca Y., Gake B., Dubot-Peres A., Grard G., Peyrefitte C. (2018). Combination of ELISA Screening and Seroneutralisation Tests to Expedite Zika Virus Seroprevalence Studies. Virol. J..

[26] Wendel S., Kutner J.M., Machado R., Fontão-Wendel R., Bub C., Fachini R., Yokoyama A., Candelaria G., Sakashita A., Achkar R. (2020). Screening for SARS-CoV-2 Antibodies in Convalescent Plasma in Brazil: Preliminary Lessons from a Voluntary Convalescent Donor Program. Transfusion.

[27] Mendrone-Junior A., Dinardo C.L., Ferreira S.C., Nishya A., Salles N.A., de Almeida Neto C., Hamasaki D.T., Facincani T., de Oliveira Alves L.B., Machado R.R.G. (2021). Correlation between SARS-COV-2 Antibody Screening by Immunoassay and Neutralizing Antibody Testing. Transfusion.

[28] Villas-Boas L.S., Paula A.V., Silva A.R.D., Paiao H.G.O., Tozetto-Mendoza T.R., Manuli E.R., Leal F.E., Ferraz A.B.C., Sabino E.C., Bierrenbach A.L. (2022). Absence of Neutralizing Antibodies against the Omicron SARS-CoV-2 Variant in Convalescent Sera from Individuals Infected with the Ancestral SARS-CoV-2 Virus or Its Gamma Variant. Clinics.

[29] Zhu Y., Chidekel A., Shaffer T.H. (2010). 3. Cultured Human Airway Epithelial Cells (Calu-3): A Model of Human Respiratory Function, Structure, and Inflammatory Responses. Crit. Care Res. Pract..

[30] Halfmann P.J., Iida S., Iwatsuki-Horimoto K., Maemura T., Kiso M., Scheaffer S.M., Darling T.L., Joshi A., Loeber S., Singh G. (2022). SARS-CoV-2 Omicron Virus Causes Attenuated Disease in Mice and Hamsters. Nature.

[31] Alberca-Custodio R.W., Faustino L.D., Gomes E., Nunes F.P.B., Siqueira M.K., Labrada A., Almeida R.R., Camara N.O.S., Fonseca D.M., Russo M. (2020). Allergen-Specific Immunotherapy with Liposome Containing CpG-ODN in Murine Model of Asthma Relies on MyD88 Signaling in Dendritic Cells Allergen-Specific Immunotherapy with Liposome Containing CpG-ODN in Murine Model of Asthma Relies on MyD88 Signaling in Dendritic Cells. Front. Immunol..

[32] Mirotti L., Custódio R.W.A., Gomes E., Rammauro F., de Araujo E.F., Calich V.L.G., Russo M. (2017). CPG-ODN Shapes Alum Adjuvant Activity Signaling via MyD88 and Il-10. Front. Immunol..

[33] Sengupta A., Azharuddin M., Cardona M.E., Devito C., von Castelmur E., Wehlin A., Pietras Z., Sunnerhagen M., Selegård R., Aili D. (2022). Intranasal Coronavirus SARS-CoV-2 Immunization with Lipid Adjuvants Provides Systemic and Mucosal Immune Response against SARS-CoV-2 S1 Spike and Nucleocapsid Protein. Vaccines.

[34] Hartmann G., Battiany J., Poeck H., Wagner M., Kerkmann M., Lubenow N., Rothenfusser S., Endres S. (2003). Rational Design of New CpG Oligonucleotides That Combine B Cell Activation with High IFN-α Induction in Plasmacytoid Dendritic Cells. Eur. J. Immunol..

[35] Vollmer J., Weeratna R., Payette P., Jurk M., Schetter C., Laucht M., Wader T., Tluk S., Liu M., Davis H.L. (2004). Characterization of Three CpG Oligodeoxynucleotide Classes with Distinct Immunostimulatory Activities. Eur. J. Immunol..

[36] Firmino-Cruz L., dos-Santos J.S., da Fonseca-Martins A.M., Oliveira-Maciel D., Guadagnini-Perez G., Roncaglia-Pereira V.A., Dumard C.H., Guedes-da-Silva F.H., Vicente Santos A.C., Alvim R.G.F. (2022). Intradermal Immunization of SARS-CoV-2 Original Strain Trimeric Spike Protein Associated to CpG and AddaS03 Adjuvants, but Not MPL, Provide Strong Humoral and Cellular Response in Mice. Vaccines.

[37] Rivera-Hernandez T., Rhyme M.S., Cork A.J., Jones S., Segui-Perez C., Brunner L., Richter J., Petrovsky N., Lawrenz M., Goldblatt D. (2020). Vaccine-Induced Th1-Type Response Protects against Invasive Group a Streptococcus Infection in the Absence of Opsonizing Antibodies. mBio.

[38] Boyaka P.N. (2017). Inducing Mucosal IgA: A Challenge for Vaccine Adjuvants and Delivery Systems. J. Immunol..

[39] Brandtzaeg P. (2009). Mucosal Immunity: Induction, Dissemination, and Effector Functions. Scand. J. Immunol..

[40] Hand T.W., Reboldi A. (2021). Annual Review of Immunology Production and Function of Immunoglobulin A. Annu. Rev. Immunol..

[41] Zhang G.F., Meng W., Chen L., Ding L., Feng J., Perez J., Ali A., Sun S., Liu Z., Huang Y. (2022). Neutralizing Antibodies to SARS-CoV-2 Variants of Concern Including Delta and Omicron in Subjects Receiving MRNA-1273, BNT162b2, and Ad26.COV2.S Vaccines. J. Med. Virol..

[42] Diallo B.K., Chasaide C.N., Wong T.Y., Schmitt P., Lee K.S., Weaver K., Miller O., Cooper M., Jazayeri S.D., Damron F.H. (2023). Intranasal COVID-19 Vaccine Induces Respiratory Memory T Cells and Protects K18-HACE Mice against SARS-CoV-2 Infection. Vaccines.

[43] Ou B.S., Picece V.C.T.M., Baillet J., Gale E.C., Powelll A.E., Saouaf O.M., Yan J., Lopez Hernandez H., Appel E.A. (2023). Nanoparticle-Conjugated TLR9 Agonists Improve the Potency, Durability, and Breadth of COVID-19 Vaccines. bioRxiv.

