# Supplementary material for: Intranasal Liposomal Formulation of Spike Protein Adjuvanted with CpG Protects and Boosts Heterologous Immunity of hACE2 Transgenic Mice to SARS-CoV-2 Infection
Source: Vaccines (Basel). 2023 Nov 20;11(11):1732. doi: 10.3390/vaccines11111732 (PMC10675295; doi:10.3390/vaccines11111732)

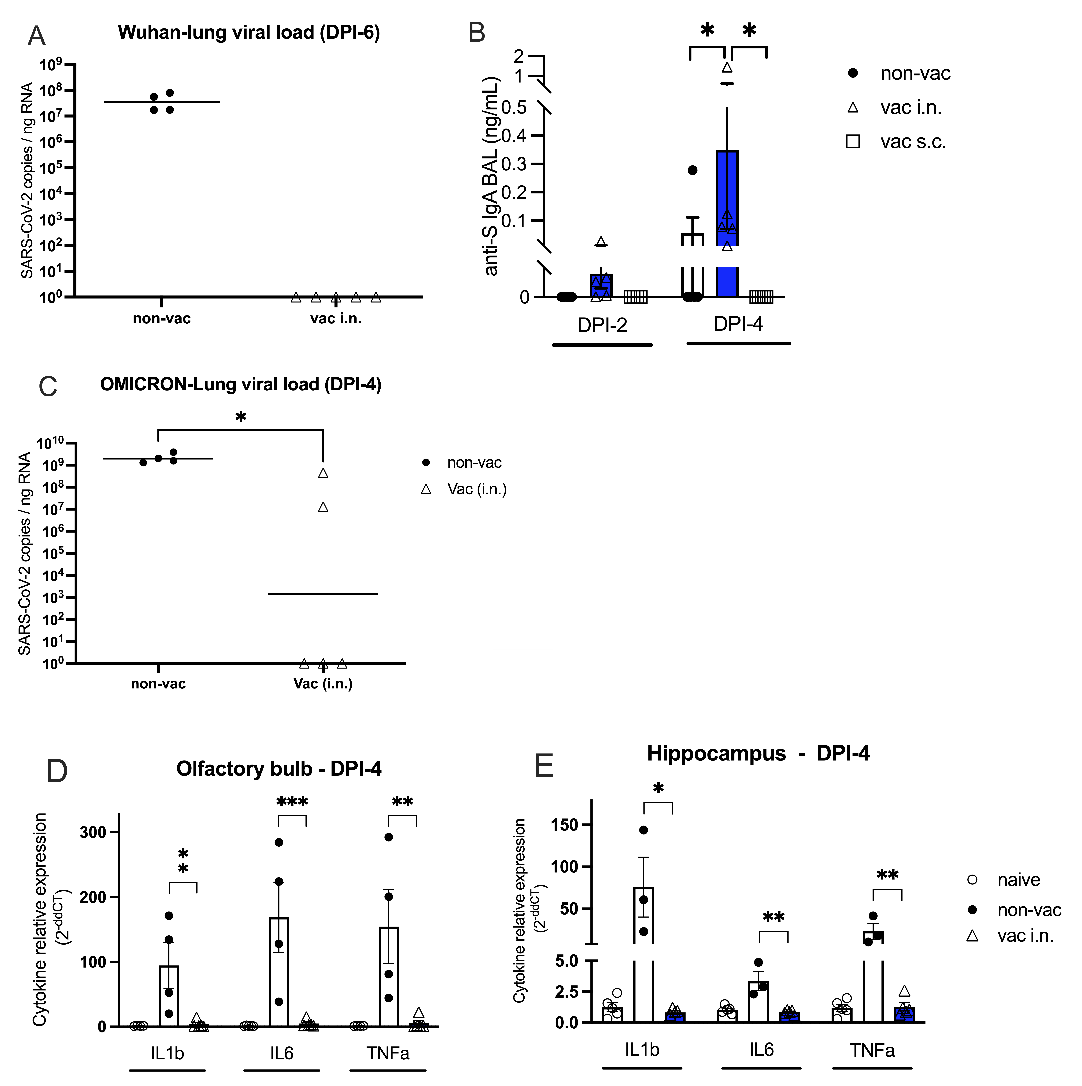


**Figure S1:** Addional data regarding figures 3 and 4. K18-hACE2 mice were vaccinated via intra-nasal (vac i.n.) with the nasal vaccine formulation on days 0 and 7. Non-vaccinated mice (non-vac) received only PBS. Mice were infected intra-nasally with Wuhan strain (A, B, D, E) or Omicron strain (C) of SARS-CoV-2, 21 days after the first dose. **(A)** mRNA was isolated from the lungs and SARS-CoV-2 was quantitated by RT-qPCR measuring spike protein gene copy number at four days after viral infection, DPI-6; **(B)** Concentration of Spike (S)-specific IgA in broncho-alveolar lavage (BAL) measured by ELISA at two and four days after viral infection, DPI2 and DPI-4 respectively; **(C)** mRNA was isolated from the lungs and SARS-CoV-2 was quantitated by RT-qPCR measuring spike protein gene copy number four days after viral infection ((DPI-4) **(D - E)** RT-qPCR relative quantification of IL-1b, IL-6, TNFa mRNA contente four days after viral infection (DPI-4); in the olfactory bulb **(D)** and in the hippocampus **(E)**. Each symbol represents one biological replicate (animal). One-way ANOVA was performed in A-D. Error bars indicate SE; * *p* < 0.05, ** *p* < 0.01, *** *p* < 0.001

**Table S1:** *In vivo* toxicity studies. Aspartate aminotransferase (AST), alanine transaminase (ALT), alkaline phosphatase (ALP) measured in blood of DC1 male and female mice, on days 0, 2, 16 and 29. Data represent av ± sd (n = number of animals).


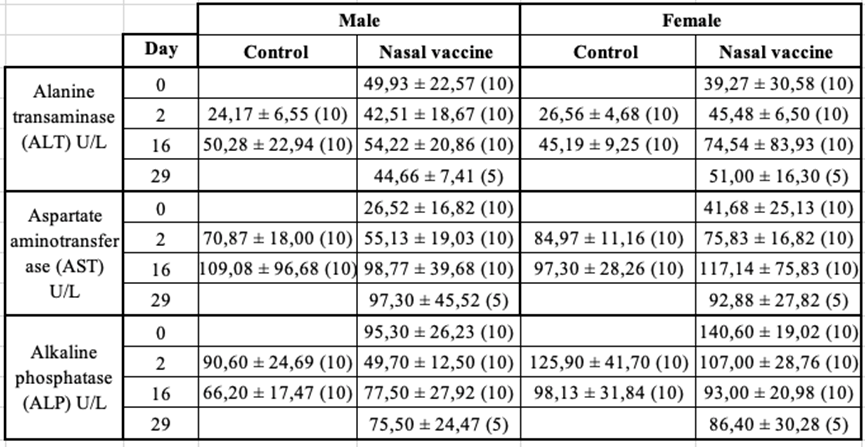

Supplement: Supplementary file 1 [file vaccines-11-01732-s001.zip › vaccines-2666146-supplementary.docx]
